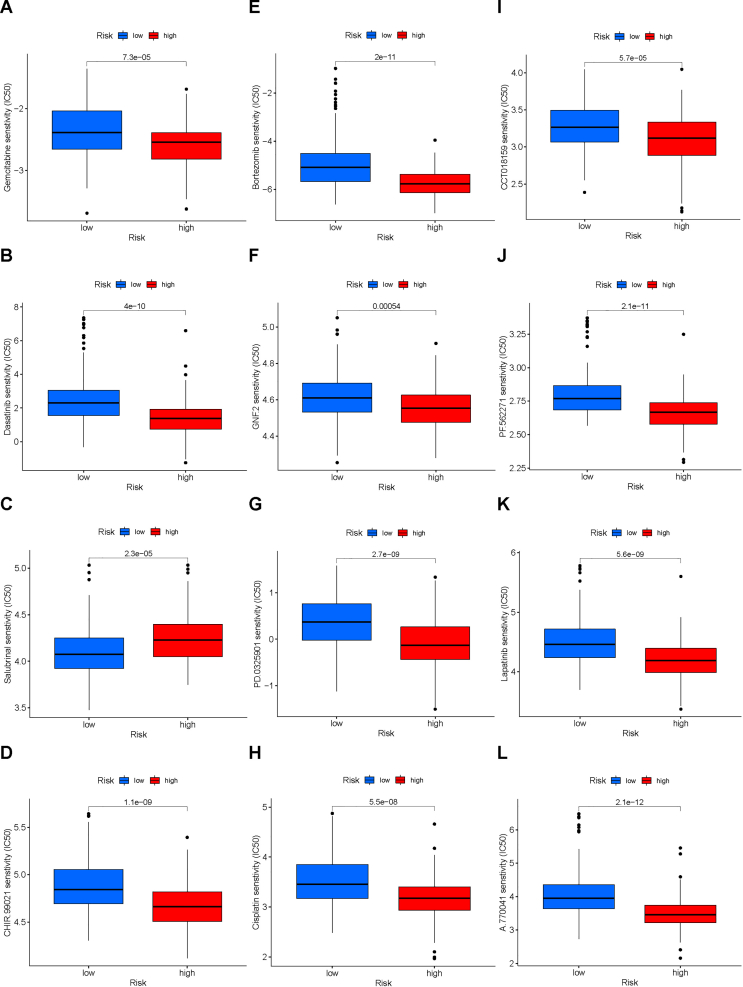# Construction of a prognostic model based on the cuproptosis-related genes in pancreatic cancer

**DOI:** 10.1016/j.gendis.2024.101391

**Published:** 2024-08-14

**Authors:** Kaili Liao, Yuxin Fu, Shuman Guo, Tingyi Qian, Feifei Teng, Yuhan Xu, Bing Sun, Hanqing Zhao, Jingyan Zhang, Ranhao Fan, Jie Gao, Xiaozhong Wang

**Affiliations:** a Jiangxi Province Key Laboratory of Immunology and Inflammation, Jiangxi Provincial Clinical Research Center for Laboratory Medicine, Department of Clinical Laboratory, The Second Affiliated Hospital, Jiangxi Medical College, Nanchang University, Nanchang, Jiangxi 330006, China; b Public Health College, Jiangxi Medical College, Nanchang University, Nanchang, Jiangxi 330006, China; c Queen Mary College, Jiangxi Medical College, Nanchang University, Nanchang, Jiangxi 330006, China; d The 1^st^ Clinical Medical College, Jiangxi Medical College, Nanchang University, Nanchang, Jiangxi 330006, China; e The 2^nd^ Clinical Medical College, Jiangxi Medical College, Nanchang University, Nanchang, Jiangxi 330006, China

Pancreatic cancer (PC) is a commonly malignant tumor with a 5-year survival rate of only 10%.^1^ Cuproptosis is a newly discovered cell death mechanism closely associated with the development of tumors. This study mainly aimed to investigate cuproptosis-related genes (CRGs) and found the marker genes to construct a prognostic model for PC patients. Meanwhile, we explored their roles in immune infiltration and their relationship with drug sensitivity. After comparing the expression patterns of ten CRGs, we found these genes were differently expressed between the tumor and normal tissues. Then we further performed functional enrichment analysis, cluster analysis, and immuno–infiltration correlation analysis. We found that cyclin-dependent kinase inhibitor 2A (CDKN2A) had the highest mutation frequency and was significantly down-regulated in tumor samples. Besides, high expression of dihydrolipoamide S-acetyltransferase (DLAT) was associated with a worse prognosis by Kaplan–Meier survival analysis. Finally, we constructed a prognostic model based on these CRGs. In the 1-year, 3-year, and 5-year receiver operator characteristic curves, the predictive accuracy of evaluation of the area under a receiver operating characteristic curve was 0.638, 0.690, and 0.796, respectively. Besides, we identified 30 potential gene mutation regulators and obtained the differences in immune microenvironment and drug sensitivity in different risk groups, which provided references for PC prediction, immunotherapy, drug therapy, and gene therapy.

We first showed the mutations of ten regulatory genes associated with cuproptosis in PC by Tsvetkov et al (2022)^2^ and conducted an in-depth analysis of the location of cuproptosis regulatory copy number variants on the chromosome (Fig. S1A–C). However, we did not find differences in the expression of these genes between tumor samples and para-carcinoma samples from the TCGA database (Fig. S1D). We supplemented 328 normal samples from the GTEX database and found that the expression of these ten cuproptosis-related genes was significantly reduced in tumor tissue (Fig. S1E). In addition, we further analyzed the expression of these genes and found that the expression of DLAT was dominantly different in the high- and low-risk groups (Fig. S1F). We noticed that the expression of DLAT regulatory factors was strongly associated with prognosis and the higher expression of DLAT meant a worse prognosis (*P* < 0.001) (Fig. S1G). Figure S1H showed the association between PRG clusters and clinical features and PRG expression in PC patients. In addition, GSVA analysis, immune infiltration, and principal component analysis results all showed significant differences between PRGclusters-A and PRGclusters-B (Fig. S1I–K). Figure S2A–D showed GO and KEGG enrichment analysis results.

ConsensusClusterPlus suggested to classify PC patients into two gene clusters (Fig. S2E). Kaplan–Meier survival analysis showed that there was no significant difference in prognosis between geneCluster-A and geneCluster-B patients (*P* > 0.05) (Fig. S2F). We also found that geneCluster-A gene mainly showed low expression of regulatory factors, but geneCluster-B gene showed high expression (Fig. S2G, H).

Next, we analyzed the differences in risk score distribution, survival status, and survival time between the high-risk and low-risk groups (Fig. S3A–I). The heatmap showed that the relative expression of three cuproptosis-related genes in all PC patients was different, and solute carrier family 16 member 1 (SLC16A1) and myoferlin (MYOF) mainly showed high expression in the high-risk group. To verify this result, we performed immunohistochemistry on pancreatic cancer tissues from stage I and stage III patients to examine the expression of SLC16A1, MYOF, and sestrin 3 (SESN3) protein, which also confirmed that SLC16A1 and MYOF were up-regulated in stage III of PC, while SESN3 was down-regulated in stage III of PC (Fig. S4). Alternatively, SLC16A1 and MYOF showed a trend toward increased expression in pancreatic cancer tissues (Fig. S5).

We used multivariate Cox regression and LASSO regression analysis to establish a risk model related to genes and screen out three genes SESN3, SLC16A1, and MYOF. Then, we used the impact diagram to display the data flow relationship among RPGcluster, geneCluster, risk, and survival status (Fig. 1A). The risk scores for all samples were counted as the following formula: risk score = − 0.2511 × exp (SESN3) + 0.4378 × exp (SLC16A1) + 0.5140 × exp (MYOF). Based on this formula, every sample was separated into the high- or low-risk group according to the median risk score. We further found the expression levels of DLAT and metal-regulatory transcription factor 1 (MTF1) at low risk were significantly lower than those at high risk, with the difference in DLAT gene being more significant (Fig. 1B). Then, 243 patient samples were randomly assigned to a training dataset (*n* = 122) and a testing dataset (*n* = 121) for Kaplan–Meier survival analysis (Fig. 1C–E). The overall survival analysis showed that the survival outcome of the high-risk group of patients was worse than that of the low-risk group. In the 1-year, 3-year, and 5-year receiver operator characteristic curves, the predictive accuracy of evaluation of the area under a receiver operating characteristic curve was 0.638, 0.690, and 0.796, respectively (Fig. 1F). To predict PC patients' survival probability, a nomogram was developed based on risk score, stage, and grade (Fig. 1G). The calibration curve reflected the relatively excellent predictive function of the nomogram (Fig. 1H). To further verify the model, we downloaded the external data set (GSE15471 and GSE16515) from the GEO database. In the receiver operator characteristic curves, both have values greater than 0.5 for the area under a receiver operating characteristic curve, which indicated that the model also had predictive capability in other datasets (Fig. 1I, J). Besides, we analyzed patient-related clinical data in the prognostic model. We found that there were no significant differences in patient risk scores at different ages and genders (Fig. S6A, B) but differences in stages and grades (Fig. S6C–G).

**Figure 1:**
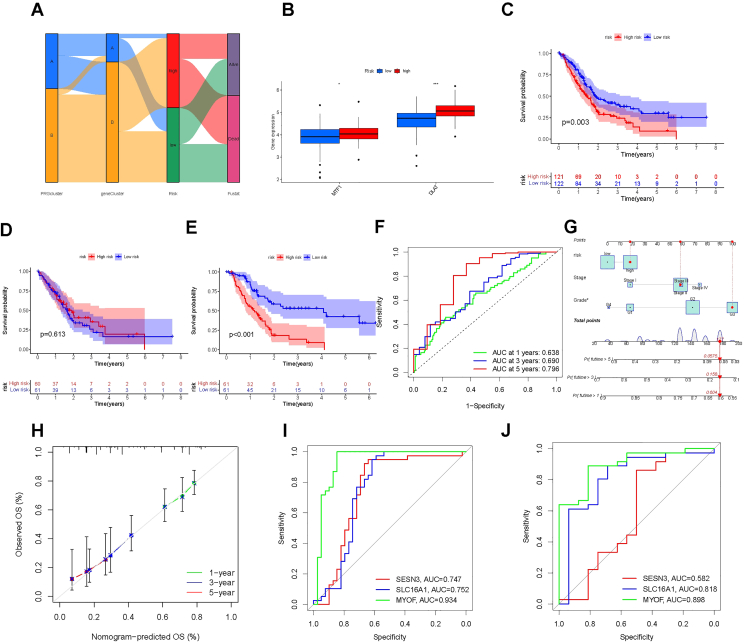
Construction and validation of the pancreatic cancer prognostic risk model. **(A)** Sankey diagram presents the relationship among PRGcluster, geneCluster, risk, and survival status in the damaged area. **(B)** The boxplot depicted the expression of MTF1 and DLAT1 in the two risk groups. **(C–E)** Kaplan–Meier survival curve showed the survival outcome of the high-risk group and low-risk group. **(F)** The receiver operating characteristic curves for predicting the sensitivity and specificity of 1-, 3-, and 5-year survival according to the cuproptosis score. **(G)** The visualization of the prognosis model in the nomogram. **(H)** Calibration curves for validating the established nomogram. *P*-values were shown as ∗*P* < 0.05, ∗∗*P* < 0.01, and ∗∗∗*P* < 0.001. **(I, J)** The receiver operating characteristic curves for model validation with external data sets (GSE15471 and GSE16515).

The bioinformatics algorithm CIBERSORT was used to estimate 22 types of tumor-infiltrating immune cells in malignant tumors. We visualized the situation of the tumor-infiltrating immune cells in high- and low-risk groups of each PC patient (Fig. S7A–F) and found risk score was significantly negatively correlated with resting memory CD4^+^ T cells, naïve B cells, resting mast cells, and monocytes, but positively associated with activated dendritic cells and activated mast cells. Besides, we examined the relationship between MYOF, SESN3, SLC16A1, and immune infiltration in PC (Fig. S7G). We visualized the relationship between tumor microenvironment (TME) scores and ESTIMATE scores, immune scores and stromal scores in different risk groups (Fig. S7H).

Features of gene mutations in PC patients are shown in Figure S8A. We analyzed the relationship between risk score and tumor mutation burden (TMB) in geneCluster A and geneCluster B (Fig. S8B). In addition, the box plot showed the differences in tumor mutation burden among different risk groups, with the tumor mutation burden of the high-risk group being higher than the low-risk group (Fig. S8C).

To guide the use of chemotherapy drugs, it was necessary to understand the degree of tumor differentiation. We analyzed the relationship between risk score and RNAss (Fig. S8D). The *P*-value was not significant and the relationship between tumor differentiation and risk score needed to be investigated. Then, using the pRRophetic algorithm, we plotted a boxplot showing the difference in sensitivity between low and high risk to IC50 semi-inhibitory concentrations of 20 chemotherapy drugs (Fig. S9A–L).

Our study systematically analyzed the CRGs in PC and established a prognostic model that performed well in predicting overall survival in PC patients, which was significantly correlated with immune infiltration level and TME expression. TME is a complex structure composed of stroma as well as cancer and immune cells. We found that the immune cell hosts involved in PC pathology are mainly naïve B cells, monocytes, and mast cells; especially, SESN3 was dominantly positively related with naïve B cells and monocytes but negatively related with mast cells. A previous study has shown an increase in the levels of mast cells in TME during the early stage of tumor development, and the high infiltration of mast cells correlates with PC progression.^3^ Downs-Canner et al found that B cells can produce granzyme B when the B cell receptor recognizes tumor cell antigens and directly kill tumor cells.^4^ Besides, monocytes can affect TME by various mechanisms and lead to immune tolerance, the proliferation of cancer cells, and angiogenesis, and trigger anti-tumor responses by activating APC.^5^ It reminded us that SESN3 can regulate these immune cells to influence the TME and achieve the anti-tumor effect, which may help in the prediction, immunotherapy, and gene therapy. There were also several limitations in our study. First, there were not enough sample sizes and data sets with clinical prognostic information for further validation, which was urgently needed in future research. Second, this study did not consider a number of other important genes with predictive value. This study had a certain reference value for the subsequent basic research on the prognosis and immunity of PC patients with cuproptosis and will provide new insights for the development of drug therapy, immune therapy, and gene therapy strategies to prevent and treat cancer.

## Ethics declaration

This study was approved by the Ethics Committee of the Second Affiliated Hospital of Nanchang University (approval numbers: (2023) CDYFYYLK (05–023)). In addition, informed consent was obtained from the patients.

## Author contributions

**Kaili Liao:** Validation, Writing – review & editing. **Yuxin Fu:** Validation, Writing – original draft. **Shuman Guo:** Visualization. **Tingyi Qian:** Validation. **Feifei Teng:** Writing – original draft. **Yuhan Xu:** Visualization. **Bing Sun:** Data curation, Formal analysis. **Hanqing Zhao:** Software. **Jingyan Zhang:** Writing – original draft. **Ranhao Fan:** Visualization. **Jie Gao:** Visualization. **Xiaozhong Wang:** Funding acquisition, Project administration, Resources, Writing – review & editing.

## Conflict of interests

There is no conflict of interests in this study.

## Funding

This study was funded by the National Natural Science Foundation of China (No. 82160405), the Jiangxi Provincial Science and Technology Department (China) (No. 20212BAG70046), the Jiangxi Provincial Science and Technology Department (China) (No. 20202BABL206019), and the Jiangxi Provincial Health Commission (China) (No. 202210346).

## Data availability

All data is available. Please contact us to access if it is needed.

## Figures and Tables

**figs1:**
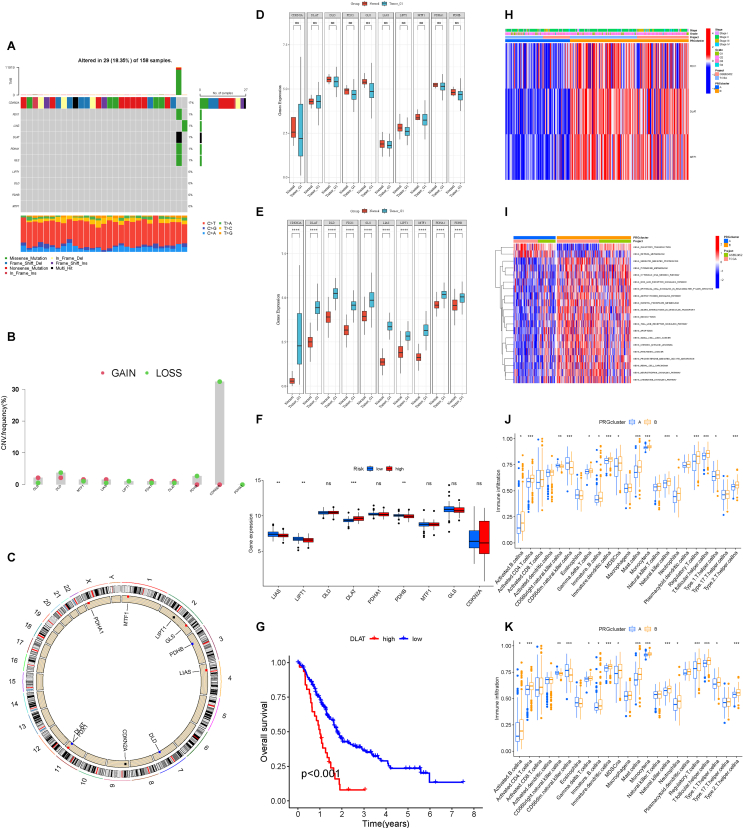


**figs2:**
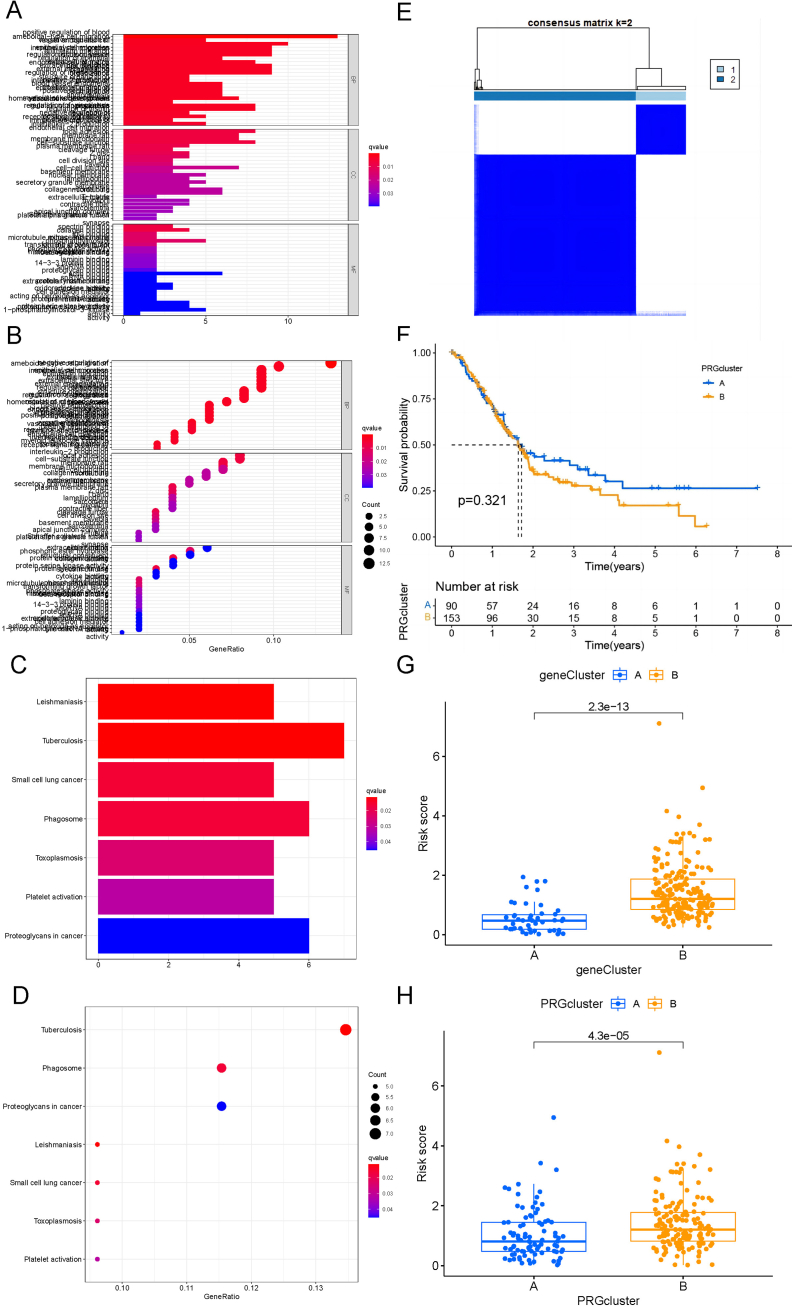


**figs3:**
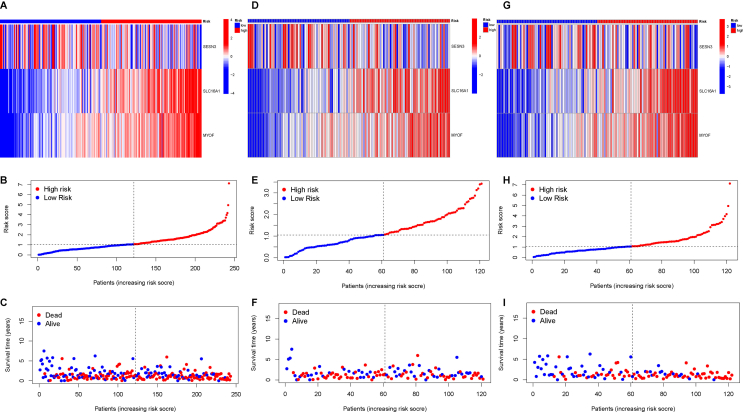


**figs4:**
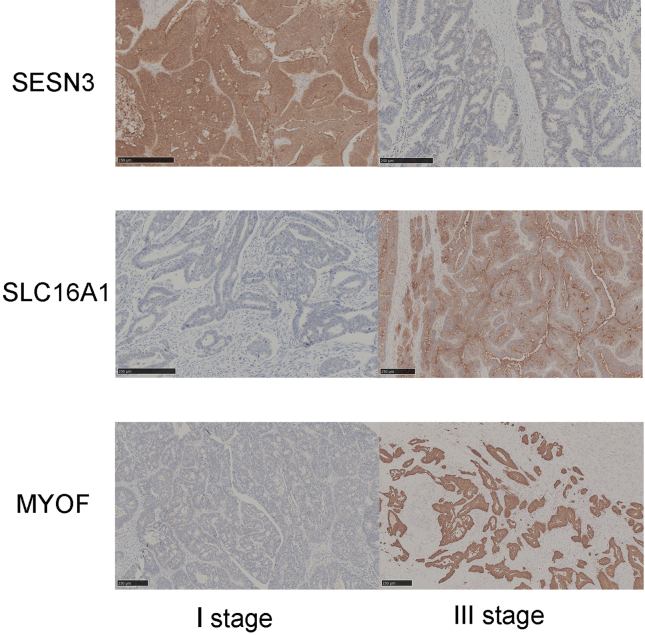


**figs5:**
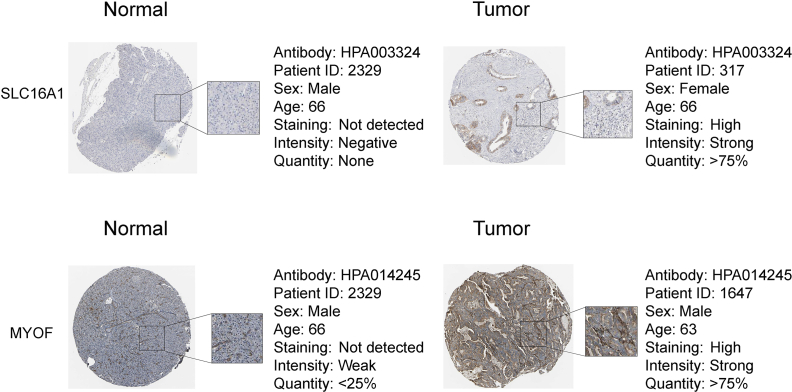


**figs6:**
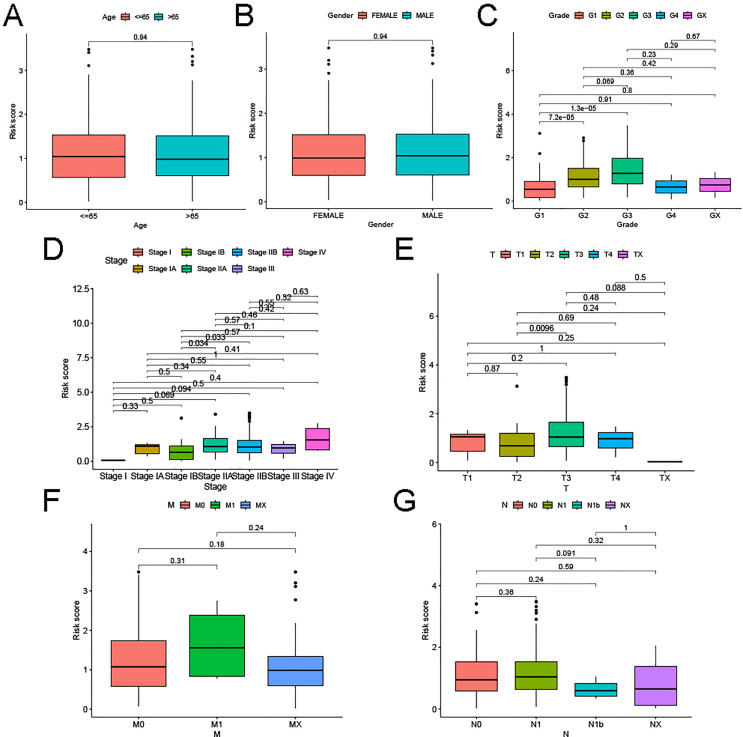


**figs7:**
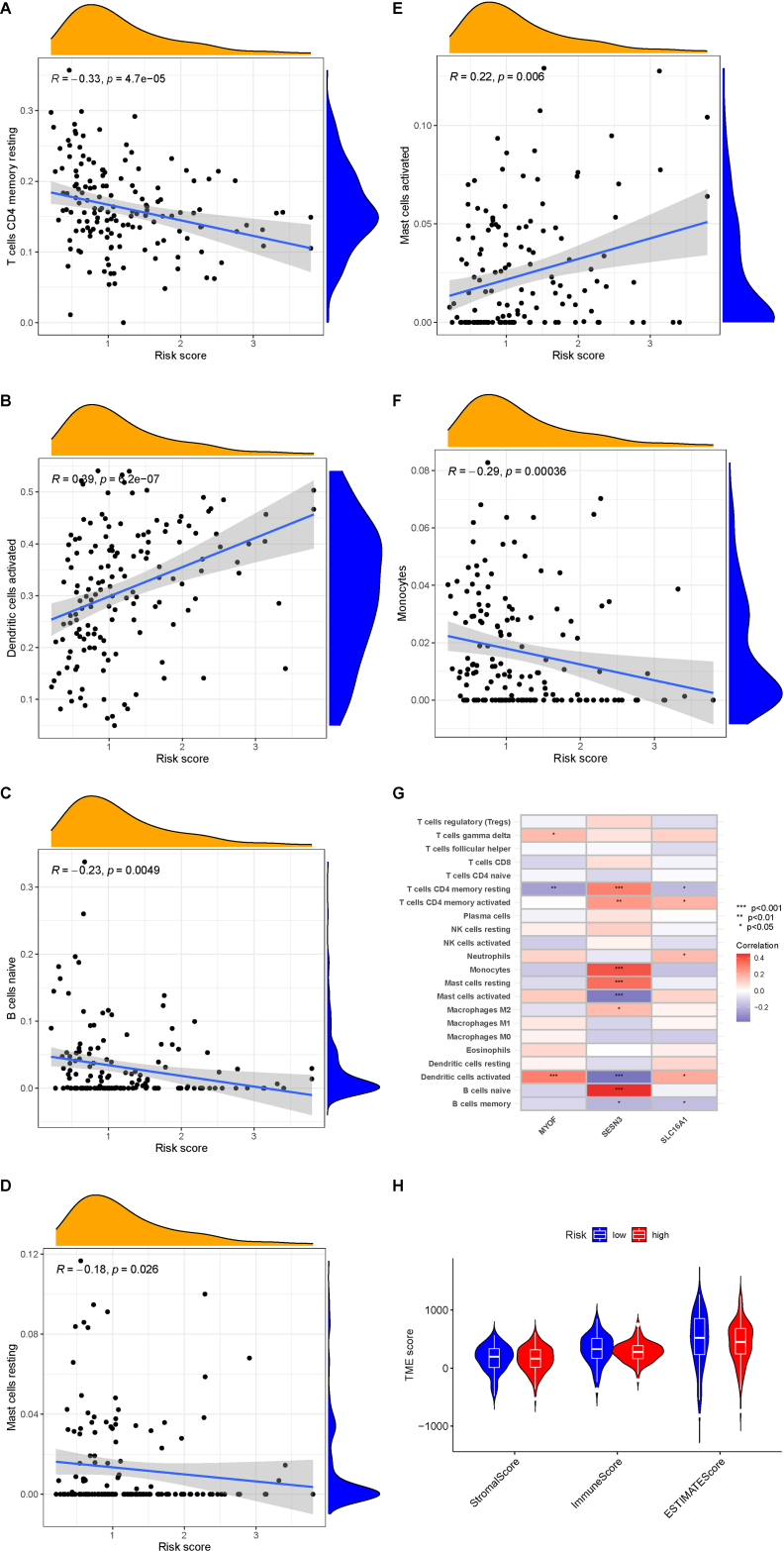


**figs8:**
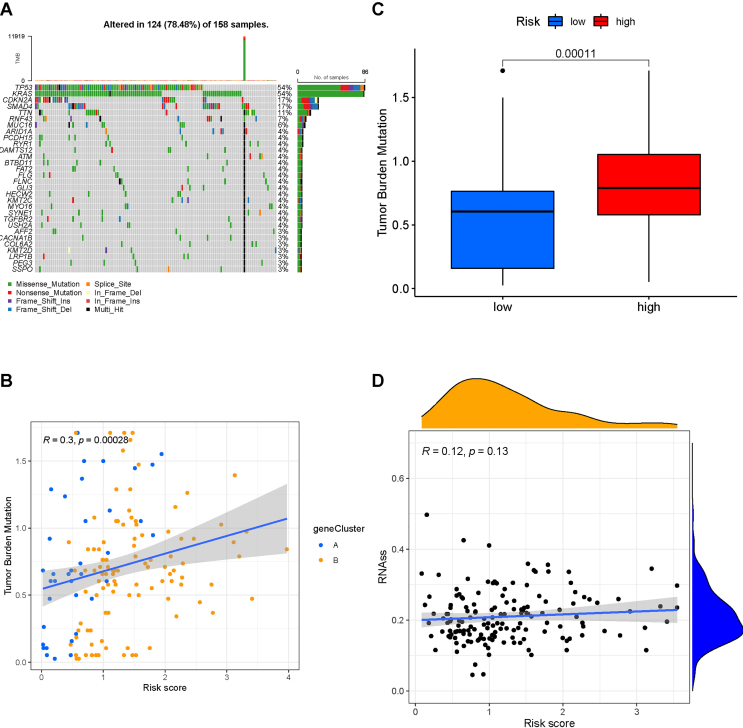


**figs9:**