# Supplementary material for: Construction of a prognostic model based on the cuproptosis-related genes in pancreatic cancer
Source: Genes Dis. 2024 Aug 14;12(3):101391. doi: 10.1016/j.gendis.2024.101391 (PMC11786824; doi:10.1016/j.gendis.2024.101391)
Supplement: Multimedia component 1 [file mmc1.docx]

**Materials & Methods**

In this study, based on 158 pancreatic cancer cell samples, we investigated the role of 10 cuproptosis related regulatory genes in pancreatic cancer, and further performed functional analysis, cluster analysis, immunoinfiltration correlation analysis and drug sensitivity analysis. Kaplan-Meier method was used to analyze the prognostic models of DLAT and MTF1 regulatory factors.

**Material collection**

In this study, 158 pancreatic Cancer cell samples were obtained from The Cancer Genome Atlas (TCGA) database and normal pancreatic tissue samples were downloaded from the GEO Series (GSE) database. We studied the role of 10 cuproptosis related regulatory genes (CDKN2A, FDX1, LIAS DLAT, PDHA1, GLS, LIPT1, DLD, PDHB, MTF1) in pancreatic cancer, and labeled the gene loci with R package "RCircos".

**The incidence of somatic mutations in cuproptosis regulators was determined**

The R package "oncoplot" was used to visualize 29 samples of pancreatic cancer cells with somatic mutations and 124 samples with mutations in 30 regulatory factors (including missense mutations, sense-free mutations, whole code mutations, frameshift mutations, and multiple hit and splicing sites). We analyzed 30 possible mutational regulators in pancreatic cancer.

**Identification and functional analysis of differentially expressed genes associated with cuproptosis**

We analyzed the location of copy number variation (CNV) of cuproptosis regulatory factors on chromosomes, and based on the results of ssGSEA expression matrix, conducted correlation analysis and drew heat map. The sample grouping was integrated with the results of ssGSEA expression matrix, and the box diagram was drawn. Using the boxplot package to visualize the range of differences between pancreatic cancer samples and normal tissue, Kaplan-Meier survival analyses were performed on 243 patient samples randomly assigned to a training dataset (N =122) and a test dataset (N =121). GO enrichment and KEGG enrichment were used for functional analysis.

**Construction of prognostic model of differentially expressed genes associated with cuproptosis**

Kaplan-Meier method was used to analyze and construct prognosis models for survival curves of DLAT and MTF1 regulatory factors. Univariate COX regression analysis was performed for overall survival (OS) to determine the presence of copper-death related differentially expressed genes associated with prognosis (pindicated statistically significant differences), and a prognostic model based on copper-death related differentially expressed genes was established. To test the accuracy of this model, we established a nomogram prediction model with multivariate Cox regression model and applied calibration method to OS results. Calibration plots showed good agreement between predicted OS and 1, 3, and 5-year survival OS, indicating accurate model construction.

**Cluster analysis**

The R package "ConsensusClusterPlus" can be used for unsupervised clustering, based on the consensus clustering method. Prognostic genes were screened by univariate Cox regression analysis. A total of 307 pancreatic cancer samples from the TCGA and GSE62452 cohorts were subtyped based on PRG and PRG, respectively. Kaplan-Meier (KM) survival analysis was performed to compare outcomes among clusters. Heat maps were used to show the correlation between PRG cluster, gene cluster and gene expression in clinical stage, clinical grade and item source, and between two clusters.

**Correlation analysis of immunoinfiltration**

For PRG cluster, R-package "GSVA" was used for single sample gene enrichment analysis (ssGSEA) to quantify the degree of immune infiltration of 23 immune cell types in the tumor microenvironment. The degree of immunoinfiltration of each immune cell type was expressed by the enrichment fraction in ssGSEA analysis, and the ranking was normalized to a uniform distribution of 0~1. We used the CIBERSORT algorithm to estimate the abundance of 22 types of invasive immune cells in high-low risk tumor samples. The correlations between the SESN3, SLC16A1 and MYOF model genes and immune infiltration were calculated using the Szpilman rank correlation coefficient: * representing a significant association prepresenting a significant association pand *** representing a significant association pEstimation of STromal and Immune cells in Malignant Tumors using Expression data The (ESTIMATE) algorithm used gene expression characteristics to estimate the ratio of stromal cells to immune cells in malignant tumor species. stromal score, immune score and ESTIMATE score were calculated by the ESTIMATE algorithm to reflect the level of immune infiltration, and then the tumor purity was deduced. Visualize the above three immune scores in different risk groups using a violin diagram.

**Correlation and drug sensitivity analysis of tumor stem cells**

We combined risk score and stemness score RNA expression-based (RNAss) to conduct Spearman correlation test. The closer the tumor stemness score is to 1, the higher the degree of stem cells and the lower the degree of differentiation. Chemotherapy response to 20 chemotherapeutic drugs in high-low risk pancreatic Cancer patients was predicted by Genomics of Drug Sensitivity in Cancer (GDSC) database. We calculated the maximum half-inhibition concentration (IC50) using the R-packet "pRRophetic" and compared IC50 differences between the high-risk groups using the Wilcoxon signed rank test, showing the results in a boxplot.

**Statistical analysis**

Differential expression genes related to cuproptosis in pancreatic cancer patients were screened by univariate Cox regression analysis. Survival analysis was performed using Kaplan-Meier and log-rank test. Spearman correlation analysis was used to evaluate the correlation between risk score and immune cell infiltration and TME score. *P*<0.05 was considered significant difference.

**Figure S1** Prognosis and tumor microenvironment characteristics in ten cuproptosis-related genes (CRGs) for pancreatic cancer (PC) patients. **(A)** Genetic alteration on a query of CRGs. **(B)** Frequencies of copy number variants (gain and loss) and non-copy number variants among CRGs. **(C)** The circus plots of chromosome distributions of CRGs. **(D, E)** The expression distributions of 10 CRGs between PC and normal tissues in the TCGA-PC and GTEX datasets. **(F)** The boxplots of the differential expression of ten genes in the high- and low-risk groups. **(G)** Kaplan-Meier curve showed the prognosis led by the expression level of DLAT. **(H)** Clinical features of the three cuproptosis genes. **(I)** GSVA enrichment analysis of biological pathways between the two distinct subtypes. **(J)** The boxplots showed the abundance of 23 infiltrating immune cell types and differences in immune scores in the two cuproptosis subtypes. **(K)** The principal component analysis showed the distribution of the two cuproptosis modification modes. *P*-values were shown as ^*^*P* < 0.05, ^**^*P* < 0.01, and ^***^*P* < 0.001. The significance of two groups of samples was determined by the Wilcoxon test, and the significance of three groups was determined by the Kruskal-Wallis test. TCGA, The Cancer Genome Atlas; PCA, principal component analysis.

**Figure S2** GO and KEGG enrichment analysis and the risk score prediction between the two clusters. **(A–D)** GO enrichment analysis ranked by adjusted *P*-value (A, B) and KEGG pathway enrichment analysis results of differentially expressed genes (DEGs) between the two cuproptosis score groups (C, D). **(E)** The consensus matrix heatmap defining two gene clusters according to the prognostic DEGs. **(F)** The Kaplan–Meier survival analysis for patients in the two PRGclusters (*P* > 0.05). **(G, H)** Differences in risk score between (G) the two gene clusters and (H) the two PRGclusters. GO, Gene Ontology; KEGG, Kyoto Encyclopedia of Genes and Genomes; BPs, biological processes; CCs, cellular components; MFs, molecular functions.

**Figure S3** Evaluation of the cuproptosis-related prognostic model in the TCGA cohort. **(A, D, G)** The heatmap showed the differential expression of genes in the cuproptosis-related prognostic model between the high- and low-risk groups in the TCGA test and train cohorts. **(B, E, H)** Distribution of the risk scores in the TCGA test and train cohorts. **(C, F, I)** The risk point plot showed the patterns of the survival time and survival status between the high- and low-risk groups in the TCGA test and train cohorts. TCGA, The Cancer Genome Atlas.

**Figure S4** The immunohistochemical results of SLC16A1, MYOF, and SESN3 in PC patients.

**Figure S5** Immunohistochemistry images obtained from the Human Protein Atlas. Selected examples of proteins found in the Human Protein Atlas database that showed trends towards differential expression in normal versus pancreatic cancer tissue.

**Figure S6** The clinical implications and prognostic role of the model in pancreatic patients. **(A–G)** Correlation between the risk score and various clinical characteristics, such as patient age, gender, and pancreatic cancer stage and grade.

**Figure S7** Correlations of the CRG score with immune infiltration and the cancer stem cell (CSC) index in pancreatic cancer. **(A–F)** Correlations between the abundance of immune cells and the risk score. **(G)** Correlations between the abundance of immune cells and three genes in the cuproptosis-related prognostic model. **(H)** Correlations between risk scores and tumor microenvironment (TME) scores. *P*-values were shown as ^*^*P* < 0.05, ^**^*P* < 0.01, and ^***^*P* < 0.001. CRG, cuproptosis-related gene.

**Figure S8** Correlations of the CRG score with TMB and ICGs in pancreatic cancer. **(A)** The mutational landscape of pancreatic cancer patients. **(B)** Correlations between the TMB and the risk score in different gene clusters. **(C)** Differences in the TMB scores between high- and low-risk groups. **(D)** Correlations between the cancer stem cell (CSC) index and the CRG score. *P*-values were shown as ^*^*P* < 0.05, ^**^*P* < 0.01, and ^***^*P* < 0.001. CRG, cuproptosis-related gene; TMB, tumor mutation burden; ICGs, immune checkpoint genes.

**Figure S9** The sensitivity of 20 chemotherapy drugs in pancreatic cancer. **(A–L)** The box plots showed the differences in chemotherapeutic sensitivity between the high-risk group and low-risk group to IC_50_ semi-inhibitory concentrations of 20 chemotherapy drugs. *P*-values were shown as ^*^*P* < 0.05, ^**^*P* < 0.01, and ^***^*P* < 0.001.
